# Effects of Radio Frequency Pretreatment on Quality of Tree Peony Seed Oils: Process Optimization and Comparison with Microwave and Roasting

**DOI:** 10.3390/foods10123062

**Published:** 2021-12-09

**Authors:** Zhi Wang, Chang Zheng, Fenghong Huang, Changsheng Liu, Ying Huang, Weijun Wang

**Affiliations:** Oil Crops Research Institute of the Chinese Academy of Agricultural Sciences, Oil Crops and Lipids Process Technology National & Local Joint Engineering Laboratory, Key Laboratory of Oilseeds Processing, Ministry of Agriculture, Hubei Key Laboratory of Lipid Chemistry and Nutrition, Wuhan 430062, China; 82101195248@caas.cn (Z.W.); zhengchang@caas.cn (C.Z.); huangfenghong@oilcrops.cn (F.H.); huangying01@caas.cn (Y.H.); Wangweijun@caas.cn (W.W.)

**Keywords:** tree peony seed oil, heating pretreatment, microstructure, volatile compounds, bioactive compounds, oxidative stability

## Abstract

In this study, we explored the technical parameters of tree peony seeds oil (TPSO) after their treatment with radio frequency (RF) at 0 °C–140 °C, and compared the results with microwave (MW) and roasted (RT) pretreatment in terms of their physicochemical properties, bioactivity (fatty acid tocopherols and phytosterols), volatile compounds and antioxidant activity of TPSO. RF (140 °C) pretreatment can effectively destroy the cell structure, substantially increasing oil yield by 15.23%. Tocopherols and phytosterols were enhanced in oil to 51.45 mg/kg and 341.35 mg/kg, respectively. In addition, antioxidant activities for 2,2-diphenyl-1-picrylhydrazyl (DPPH) and ferric-reducing antioxidant power (FRAP) were significantly improved by 33.26 μmol TE/100 g and 65.84 μmol TE/100 g, respectively (*p* < 0.05). The induction period (IP) value increased by 4.04 times. These results are similar to those of the MW pretreatment. The contents of aromatic compounds were significantly increased, resulting in improved flavors and aromas (roasted, nutty), by RF, MW and RT pretreatments. The three pretreatments significantly enhanced the antioxidant capacities and oxidative stabilities (*p* < 0.05). The current findings reveal RF to be a potential pretreatment for application in the industrial production of TPSO.

## 1. Introduction

The peony (*Paeonia Suffruticosa*) has been a popular traditional ornamental and medicinal plant in China for 2000 years [1]. More recently, tree peony has been widely cultivated for the high oil yield rate of its seeds (>27%) and because the protein content of the meal reaches 32.44% [2]. The high level of interest in tree peony seed oil (TPSO) might be explained by its abundant unsaturated fatty acids (UFAs, >90%), high proportions of α-linolenic acid (C18:3, ALA, >40%) and richness in sterols (55.00%), γ-tocopherol (average 362.98 mg/kg) and phenolic compounds (ranging from 49.16 to 130.59 mg/kg) [3,4,5]. These nutrition compounds have many physiological functions, which can exert antidiabetic, antihyperlipidemic, serum cholesterol lowering and antitumor effects [6,7,8].

Oil is usually extracted from seeds by pressing, solvent, aqueous enzymatic, supercritical CO_2_ extraction, and microwave-assisted methods. The most commonly used methods to extract oils are solvent extraction (SE) and hot pressing. SE is the most efficient method, and produces less residual oil but is environmentally unfriendly. Oil extraction by hot-pressing is simpler, safer, and has fewer steps than SE [9], but these two methods produce poor quality oil due to high processing temperatures and a relatively high number of processing steps. Its high cost on an industrial scale has limited the development of aqueous enzymatic and supercritical CO_2_ extraction processing. Cold pressing has been developed given the demand for natural green food, and is used in rapeseed oil [9], camellia seed oil, and linseed oil [10], to effectively preserve the nutrients in oils. However, the maximum oil yield of TPSO through cold pressing is only 23.11% [11]. Suitable heating pretreatments should provide efficient technologies for producing high-quality oils, and have various effects such as the promotion of oil solubility, increasing the level of bioactive components, and improving flavor. Currently studied technologies include autoclave, cooking, oven-heating, roasting (RT), steam explosion, microwave (MW) and radio frequency (RF) [12].

RF and MW are electromagnetic-field-based heating technologies. Compared to RT (convection heating), dielectric heating can penetrate the inside of seeds and provide fast and volumetric heating effects. In contrast to MW (2450 MHz), RF (27.12 MHz) has a longer wavelength, which can hasten the rate of drying, increase the quantity, and ensure heating is more uniform [13]. RT, as a traditional technology, is a simple and accessible pretreatment that is generally performed at temperatures of 100–200 °C, with a high-temperature and a long time process. A previous study revealed that RT treatment compromises several of the physicochemical properties of chia seeds [14]. Excessive RT heating of TPSO produces high contents of benzoic acid [15], which results in poor oil quality. Microwave heating, which was considered to be a new high-efficiency and promising technology has been widely used in many processes, including in the processing of rapeseed and camellia seed, to improve the texture and the release of nutrient components and enhance its antioxidant effects [16,17]. Longer nutrient retention was observed in the rapid conversion of electromagnetic energy to thermal energy in MW heating than for conventional RT [18]. RF heating is mainly used to control pests and fungi in diverse grains, nuts and beans, and is widely used for heating, drying dis-infestations, and pasteurization [19]. Recently, RF was proven to be able to extract pectin, phenolic compounds, and anthocyanins [20]. Ruange Lana et al. [21] showed that RF heating could be an efficient method for heterogeneous food matrices. Conversely, there is no information in the literature on the application of RF as a pretreatment of oilseeds to enhance the quality of TPSO.

Previous studies have mostly focused on exploring the influence of different varieties and genotypes of tree peony seeds (TPS) on oil quality [8,22,23]. However, the product of oil processing technology has not been discussed, and few researchers have studied the effect of heating pretreatment on the quality of TPSO. Therefore, the objectives of this study were to optimize the operating conditions involved in the RF assisted TPS extraction of TPSO in achieving efficient extraction, and to compare the physicochemical properties, bioactivity, volatile compounds and antioxidant activity of TPSO extracted by RF, MW and RT using the same operating conditions.

## 2. Materials and Methods

### 2.1. Materials and Reagents

Fresh tree peony seeds were obtained from Heze (Shandong, China). The moisture content was 4.78%. The adjustment of moisture content of 8% was decided after the preliminary experimental study of the effect of different moisture (6%, 7%, 8%, 9%, 10%) (Supplement Table S1) on oil extracted from the seeds at RF 140 °C, for which it was found that 8% led to a higher oil yield.

The 5α-cholestane (purity ≥ 97%), α-tocopherol, β-tocopherol, γ-tocopherol, δ-tocopherol, 37 fatty acid methyl ester mixtures standard, BSTFA + TMCS, squalene, DPPH, TPTZ, FeCl_3_, KBr, Trolox and p-anisidine were purchased from Sigma-Aldrich (St. Louis, MO, USA). Chromatographic grade reagents were purchased from Merck (Darmstadt, Germany). The analytical reagents were purchased from Sinopharm Chemical Reagent Co., Ltd. (Beijing, China). 

### 2.2. Pretreatment and Preparation of the Samples

Radio-frequency pretreatment (with hot-air) was conducted using the GJD-2B-27II-JY (Huashi Jiyuan high-frequency equipment Co., Ltd., Hebei, China). TPS (weight 500 ± 0.5 g) were evenly spread in 150 mm-in-diameter Pyrex Petri dishes, with electrode gaps of 70 mm, and a cavity temperature set to 80 °C. The seeds were treated with different RF temperatures (0 °C, 80 °C, 100 °C, 120 °C and 140 °C).

For the microwave treatment, 500 g of seed-kernels was placed in 8 Pyrex Petri dishes (9 cm diameter) and conditions were set at 800 W, 5 min (140 ± 5 °C) and a frequency of 2450 MHz inside a microwave (Model Mars, 800 W, CEM, Charlotte, NC, USA).

For the roasting treatment, TPS (500 g, kernel) on aluminum foil were oven-roasted at 140 °C for 20 min. Each condition was repeated for triplicate tests. 

### 2.3. Cold Pressing

TPS was pressed with a cold-press machine (CA59, Monchengladbach, Germany), and centrifuged at 10,000 rpm for 20 min (Sorvall Stratos, Thermo Fisher Co., Ltd., Waltham, MA, USA). All samples were placed in amber glass bottles and were sealed and stored in a refrigerator (4 ± 2 °C). The oil yield was calculated following the method of Cong et al. [24].

### 2.4. Fourier-Transform Infrared Spectroscopy (FTIR)

The sample was mixed with potassium bromide (KBr) at a mass ratio of 1:30 for tableting. The infrared spectra of the gels were detected from 4000 to 400 cm^−1^ at a 4 cm^−1^ resolution using the Fourier transform infrared (FT-IR) spectra, and recorded on a TENSOR 27 FTIR spectrometer (Bruker, Karlsruhe, Germany).

### 2.5. Transmission Electron Microscopy (TEM)

The seed sample was cut to a size of less than 1 mm^3^. The sample was completely immersed in a 2.5% glutaraldehyde solution and 0.1 M phosphate buffer solution and fixed for 4 h. A 0.1 M phosphate buffer solution was used for washing for 15 min for 3 repetitions, then fixed in a 1% osmium acid solution for 1–1.5 h. This was repeated with a 0.1 M phosphate buffer solution, with washing performed 3 times, for 15 min each. The sample was dehydrated with an acetone solution at gradient concentrations. Then, the sample was embedded in Epon 812 resin. A pure acetone and an embedding solution were placed at room temperature for 0.5 h, and 37 °C for 1.5 h, respectively. Finally, the sample was cured at 37, 45, or 60 °C for 24 h. A 70 mm slice was obtained using a Reichert-Jung UL TRACUT E ultrathin slicer (Leica, Vienna, Austria), and was dyed with a lead citrate solution and uranyl acetate for 15 min, then observed under a JEM 1200 transmission electron microscope (JEOL, Tokyo, Japan). 

### 2.6. Determination of Physical and Chemical Properties

The acid value (AV) was measured according to GB 5009.229-2016 [25]. For the determination of color, we used a PFXi-Series automatic lovbind tintometer (PFXI 995, German) to evaluate the CIE (L*, a*, b*) of samples. The following parameters were determined: lightness (L*), redness+/greenness (a*), and yellowness+/blueness (b*). The total phenolic content (TPC) was determined using the method described by Cong et al. [26]. The results are expressed as gallic acid equivalents (mg GAE/100 g). The carotenoid content of the oils was determined according to the MPOB (2005) standard [27].

### 2.7. Determination of the Tocopherols Content 

Tocopherols contents were measured according to the AOCS Official Method Ce 8-89 [28]. The HPLC conditions were the same as those reported by Cong et al. [26].

### 2.8. Determination of Fatty Acids Profile

The fatty acids content was determined according to GB5009.168-2016 [25]. Briefly, we placed three drops of TPSO in a 10 mL centrifuge tube, adding 0.5 mL (0.5 mol/L) sodium methoxide and 2.5 mL n-hexane, after which the supernatant was injected into a GC (GC7890A, Agilent, CA, USA) equipped with a flame ionization detector (FID) to quantify fatty acid methyl esters. The column was DB-FFAP (30 m × 250 μm id × 0.25 μm thickness). The temperatures of the injector and detector were both set to 300 °C, the flow rate of nitrogen was 1.5 mL/min with a split ratio of 80:1. The column temperature was maintained at 210 °C for 9 min, then it was increased by 20 °C/min to 250 °C and maintained for 10 min.

### 2.9. Determination of the Phytosterols and Squalene

For saponifying 200 mg of oil, we added 0.1 mL of 1 mg/mL 5α-cholestane as the internal standard, then 2 mL of 2 mol/L KOH-Ethanol was added to the sample, and the oil samples were placed in a saponified bath at 70 °C for 60 min. After saponification was completed, we added 1.5 mL of distilled water and 1 mL of hexane to the sample. The extraction was repeated three times. Following this, the mixture was centrifuged at 4000 rpm for 10 min. Then, we transferred the sample to a 2 mL injection bottle. After the removal of hexane by nitrogen blowing, 100 μL of BSTFA + TMCS was added to the glass tube containing the injection bottle and the mixture was allowed to react in an oven at 105 °C for 15 min. Finally, we used n-hexane to increase the volume to 1 mL for analysis. 

A 1 μL derivative sample, with a resolution ratio of 20:1, was injected into an Agilent GC7890A and 7683B series equipped with an FID, and a silica gel capillary column (DB-5MS, 30 m × 0.25 mm × 0.25 μm; Agilent, Santa Clara, CA, USA). The flow rate was 1.2 mL/min, and helium was used as the carrier gas. The temperature of the injector and detector were both set to 290 °C. The column was kept at 100 °C for 1 min. The column temperature was increased from 60 °C to 290 °C at a rate of 40 °C/min and maintained for 15 min. The sterol was identified via a comparison with the retention time of the standard, and quantified based on the relative peak area of the internal standard. The GC-MS ion source temperature was set to 290 °C, and the transmission line was set to 290 °C in the SIM monitoring mode. An electron impact mass spectrum was generated at 70 eV. The scanning range was *m*/*z* 50–500.

### 2.10. Sensory Evaluation 

The sensory evaluation was carried out by 8 trained members between the ages of 25 and 40 from the Institute of Oil Crops Research. Each team member received one week of TPSO assessment training. TPSOs were produced through different pretreatments methods. The team members compared the aroma parameters (roasted, nuts, fat, green, sweetness and pungency), and assigned a score to each sample based on the odor intensity for a sensory evaluation of the aroma of peony oil, with a score range of 0–10, where 0–3 indicates that the odor is very weak and difficult to distinguish, 3–7 means that the odor is clear and the intensity is low, and 8–10 means that the odor is clear and strong. At the same time, the overall scent degree was measured using a score range of 0–10 points.

### 2.11. Determination of Volatile Compounds

Volatile compounds were determined by headspace solid-phase micro-extraction (HS-SPME) according to Xiao et al. [29]. The condition of extraction and the GC-MS conditions were slightly different. The oil was equilibrated at 60 °C for 20 min through an automatic SPME module (Agilent, CTC G6500, Santa Clara, CA, USA) and the volatile compounds were automatically extracted for 30 min.

The Agilent 7890A GC and 5975C series mass spectrometers (GC-MS, Agilent, Santa Clara, CA, USA) were used to identify the volatiles in the TPSO extracted by the three different pretreatment methods. The extracted compounds were separated in polar DB-WAX (30 m × 320 μm × 0.25 μm). The working conditions were 250 °C in split mode with an inlet temperature of 1:10. The carrier gas was helium (99.999%), and the flow rate was 1.5 mL/min. The operating conditions were as follows: heating at 40 °C for 2 min, heating at 5 °C/min to 200 °C, and maintained for 2 min, then heating at 8 °C/min to 250 °C, and finally, maintaining this temperature for 5 min. The GC-MS transfer line was set to 280 °C; an electron impact mass spectrum was generated at 70 eV. The scanning range was *m*/*z* 38–400. All compounds were identified according to the NIST 2017 (Qual ≥ 80) mass spectrometry library and reliable standards. 

### 2.12. Determination of the Oxidative Stabilities of the Oils

The peroxide value (POV) was measured according to GB 5009.227–2016 [24]. For K_232_ (CD) and K_270_ (CT), absorbance at 232 (K_232_) and 270 (K_270_) nm was measured according to the ISO standard 3656:2002 [30]. P-anisidine value (P-AV) was measured according to the AOCS Official Method Cd18-90 [31]. The induction period (IP) was determined according to the method of Azadmard et al. [16].

### 2.13. Determination of the Antioxidant Activity of the Oils

The 2,2-diphenyl-1-picrylhydrazyl (DPPH) and ferric-reducing antioxidant power (FRAP) were measured following the method of Cong et al. [26]. The DPPH and FRAP results are expressed as micromoles of Trolox equivalents per 100 g of sample (μmol TE/100 g).

### 2.14. Statistical Analysis

The experiment data were expressed as the mean value ± standard deviation from the triplicate tests (3 replicates per treatment). SPSS 20.0 (SPSS Inc., Chicago, IL, USA) was used to perform the ANOVA and Duncan’s multiple range test (*p* < 0.05). All figures were drawn in Origin 9.0 software (Origin Lab Corp., Northampton, England).

## 3. Results

### 3.1. Different Pretreatments Seed Microstructure (FTIR and TEM) 

**Figure 1:**
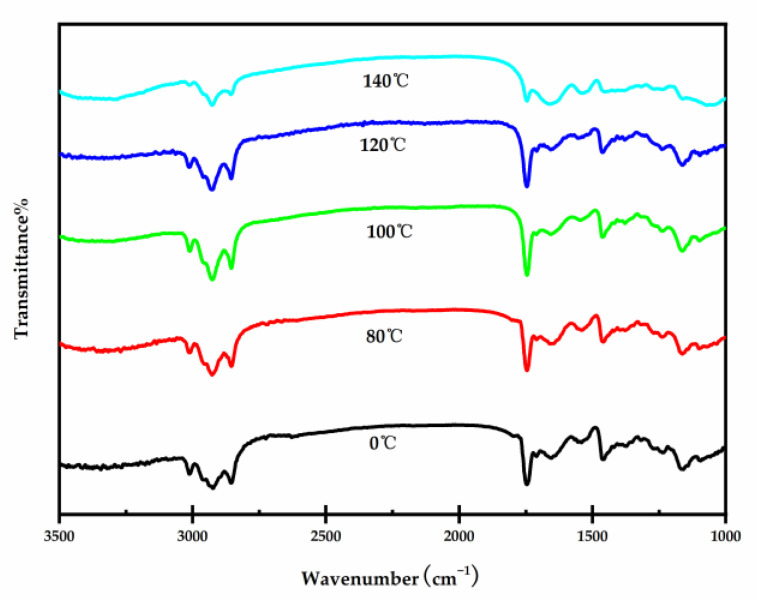
FTIR spectra of untreated seeds (0 °C) and of the different radio frequency pretreatment seed samples treated at a temperature of 80, 100, 120 and 140 °C.

Figure 1 displays the main peaks of the FTIR spectrum and the TPS of the different temperatures of RF groups. The FTIR spectrum can be classified into 7 groups: (1) 3000–3600 cm^−1^, (2) 3282 cm^−1^, (3) 2800–3000 cm^−1^, (4) 1745 cm^−1^, (5) 1645–1647 cm^−1^, (6) 1500–1600 cm^−1^ (7) 1246 cm^−1^. The wave number range of 3000–3600 cm^−1^ was a result of the broadband center and was attributed to potential hydrogen bond interactions (OH and NH groups) and hydrogen bond stretching vibrations of cis-olefinic double-bonds. The existence of peaks in the wave number range of 3282 cm^−1^ indicates the band assignments of the ν (=C–H) and OH groups vibrating strongly. The existence of peaks in the wave number range of the lipid band area 2800–3000 cm^−1^ indicates a characteristic of the stretching C–H bond in methyl groups. The 1745 cm^−1^ peak is associated with C=O bonding, conjugated bonds of triglycerides, bending and the stretching vibrations of aliphatic groups of triglycerides. The observed peaks in 1645–1647 cm^−1^, 1500–1600 cm^−1^, 1246 cm^−1^ are probably due to the existence of amide I (C=O bonding), amide II, amide III in the structure, respectively [32,33]. The visual investigation of the IR spectra of TPS treated by different temperatures does not show any marked difference. While comparing peak intensities, a slight change was noticed in certain IR spectral peaks of TPS of different temperatures. FTIR spectroscopy was applied to investigate the inter molecular interactions in protein and polysaccharide matrices [32]. The TPS at the RF of different temperatures showed a decrease in the intensity of peak at 1745 cm^−1^ compared to those that were untreated. The reason could be related to the polysaccharides (carbonyl group) of the seed sample decreasing with increasing temperatures. Similar results were observed in the Fourier transform infrared spectrum of chia seed flours [34]. The intensity of the peaks at 1645 cm^−1^ and 2800–3000 cm^−1^ decreased in the samples, which may be due to a decrease in the triglycerides and protein contents. Zhong et al. [35] reported similar results of a reduction in lipid and protein contents (%) after RF treatment. The peaks in the FTIR spectrum could also be related to the creation of melanoidins during the RF heating process [33].

**Figure 2:**
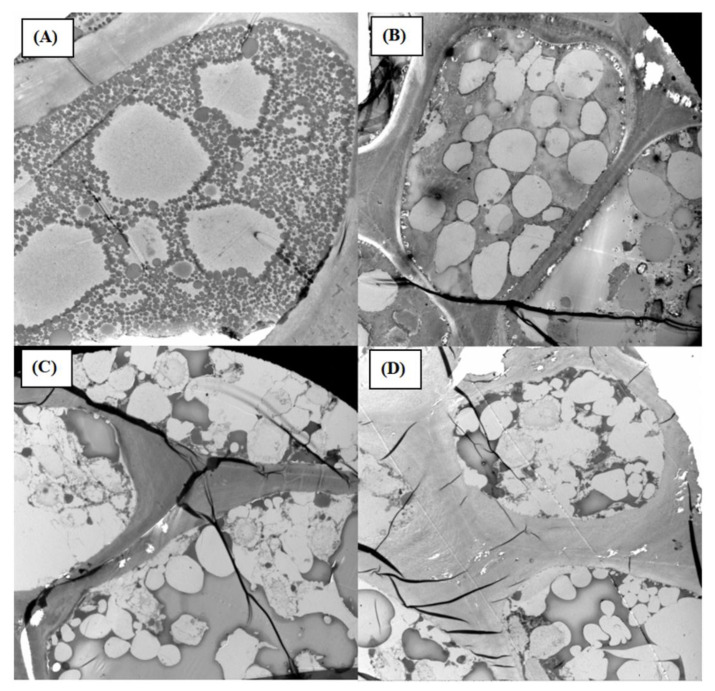
TEM observations of seeds subjected to different pretreatments. (**A**) untreated; (**B**) RF (radio frequency) at 140 °C; (**C**) MW (microwave) at 140 °C; (**D**) RT (roasted) at 140 °C.

Figure 2 shows the TEM results of the internal structure of the TPS, of that which was untreated, RF140 °C, MW140 °C and RT140 °C. The results show that the destruction of the cell structure formed irregular cavities and evident porosity after heating through RF, MW and RT pretreatments. Compared to those that were untreated, those treated by RF TPS showed structural changes in the protein and lipid bodies in the cells, and no intact cells were observed. The cellular bodies were ruptured to release the oil, which gathered into droplets and sufficiently interacted with oil, with the droplets finally coalescing. Wroniak et al. [36] suggested that MW can reduce the moisture content and increase cell rupture during pressing, ultimately achieving the objective of increasing oil extractability. The reason for the increase in oil yield was that MW has high-frequency electromagnetic waves, which can be quickly converted into heat energy, leading to cell lysis [17]. The principle of RF was similar to that of MW. RF pretreatment at 140 °C could also damage the cell structure. According to the TEM images, the cell wall was highly likely to be severely damaged, as a result of which the oil would be released from damaged cells to achieve a high free oil yield. All three pretreatment technologies can, to some extent, destroy the cell structure.

### 3.2. Physicochemical Properties of Tree Peony Seed Oil

In this study, the oil content of the seed was 34.55%. The physical and chemical properties of TPS treated by RF at different temperatures (80, 100, 120 and 140 °C) are presented in Table 1. The effects on the oil yield were firstly investigated at different temperatures of RF. The oil yield significantly increased after different temperatures (*p* < 0.05), ranging from 15.57% (untreated) to 30.80% (RF at 140 °C), which was 1.98 times higher than that of the untreated TPS. As the temperature increased in RF treatments, the acid value (AV), TPC, and content of total carotenoids increased (Table 1). More specifically, the acid value (AV) ranged from 4.07 ± 0.04 (mg/g) (for untreated) to 5.88 ± 0.01 (mg/g) (at RF treatments140 °C), the total phenolic content ranged from 4.78 ± 0.01 (mg GAE/100 g) (for untreated) to 54.12 ± 0.06 (mg GAE/100 g) (RF treatments at 140 °C), and the β-carotenoid content ranged from 0.41 ± 0.02 (mg/kg) (for untreated) to 0.62 ± 0.14 (mg/kg) (at RF treatments140 °C). Both AV and TPC increased drastically at 120 °C, the initial acid value of TPSO was relatively high, and the deacidification process needed to be considered. 

Then, we compared the physicochemical properties of TPSO extracted by different pretreatment (RF, MV, RT) methods at 140 °C. The oil yield treated with RF, MW and RT increased by 15.23%, 16.32% and 14.71%, respectively. The reason for this finding is that the cell membrane and cell wall of the seed easily rupture, so oil was released after heating treatment. Hu et al. [37] studied MV-assisted oil extraction from tea seed, and the oil yield was increased by 7.13% compared to the oil that was untreated. This result was consistent with the data in this paper. RF proved to be an effective pretreatment method to increase the oil output rate. The L* was significantly decreased at 140 °C for all three pretreatments (*p* < 0.05). Ling et al. [38] used different pistachios pretreatments, whereby the L* of oil was darker after MW and RT. A similar trend was observed in this study in the L* value. The a* value of TPSO treated at different RF temperatures increased significantly, darkening the sample (reddish). The darkening was probably due to caramelization and Maillard reactions and other chemical reactions [39]. Among the three different pretreatments methods, RT resulted in the most significant change, followed by MW and RF. Carotenoid particularly affected the b* value (yellow pigment) of vegetable oil (significantly correlated at the 0.05 level (r = 0.541). After RF, MW and RT, the TPCs increased significantly by 11.42, 13.4 and 12.9 times, respectively. This may be due to the bond between polyphenols and protein being broken.

### 3.3. Fatty Acid Compositions, Tocopherols and Phytosterols in TPSO 

The bioactive properties of TPSO, produced by the different temperatures of RF, were investigated, and the results of MW and RT at 140 °C were compared with the untreated oil obtained from untreated seed samples. The fatty acid composition, the tocopherol and phytosterol contents of TPSO produced through the three heating treatments of TPS are presented in Table 2. The most abundant fatty acids were palmitic (16:0), oleic (18:1), linoleic acids (18:2) and α-linolenic acid (18:3). The fatty acid content remained at 5.64–5.73% (16:0), 22.32–22.91% (18:1), and 24.01–24.65% (18:2) and 45.27–46.06% (18:3), respectively. The results showed that unsaturated fatty acids (UFAs) were above 92%, of which the content of α-linolenic acid (ALA, C18:3) was the highest, at above 45%. ALA is an essential fatty acid that cannot be synthesized by humans and must be obtained through consumption. TPSO shows potential as a resource. The α-linoleic acid levels were slightly reduced in oil after RF, MW, RT pretreatments at 140 °C, decreasing by 1.73%, 0.40% and 0.69%, respectively. The saturated fatty acids (SFA, 16:0 and 18:0) contents were 7.17–7.85% for TPSOs. We observed no significant difference in the 0.05 levels of UFAs and SFAs after RF, MV, RT pretreatments at 140 °C. Different types of UFAs in TPSO can lower blood cholesterol and are closely related to human health [40].

The contents of tocopherols in oil are shown in Table 2. The most abundant tocopherol in the TPSO samples was γ-tocopherol, followed by α- and δ-tocopherols for which the contents were relatively low. Firstly, at different RF temperatures, findings revealed that as the RF temperature increased, the amount of total tocopherol increased significantly, at 6.5% higher than the initial value. More specifically, the γ-tocopherol content varied between 714.61 mg/kg (untreated) and 778.76 mg/kg (RF at 140 °C). The δ-tocopherol content was between 6.40 mg/kg (untreated) and 9.51 mg/kg (RF at 140 °C). In addition, the α-tocopherols content was slightly reduced in TPSO, determined to be between 45.96 mg/kg (RF at 140 °C) and 51.76 mg/kg (control). Additionally, by preheating using the RF, MW and RT pretreatments, at 140 °C, the γ-tocopherol content was significantly increased to 778.76, 761.74 and 750.44 mg/kg, respectively. However, the same technology caused a significant (*p* < 0.05) reduction in α-tocopherol contents by 5.80, 1.78 and 7.2 mg/kg, respectively. There was no significant difference (*p* < 0.05) in the total content. Tocopherols contents in TPSO were high, which could prevent the high contents of UFAs from oxidation, γ-tocopherol showed a better antioxidant ability, decreasing the risk of cancer, and performing anti-inflammatory activities [41,42].

A statistical analysis showed that the contents of the seven phytosterols and squalene TPSOs produced by different pretreatments varied significantly (*p* < 0.05). The phytosterol with the highest content of TPSO was β-sitosterol (1454.75 ± 4.16 mg/kg), followed by fucosterol, which was a unique plant sterol in TPSO, since a similar distribution was rarely reported in other vegetable oils. The phytosterols compounds in this study were, to a limited extent, similar to the findings reported by Chang et al. [5]. Of the different RF temperatures, the highest squalene content was 15.62 mg/kg at 140 °C. The content of β-sitosterol in the different groups ranged from 1454.75 mg/kg to 1645.67 mg/kg, fucosterol ranged from 677.24 mg/kg to 705.75 mg/kg, which increased by 190.92 mg/kg and 28.51 mg/kg respectively, followed by Δ-5-avenasterol and 24-methylenecycloartan-3β-ol, which increased from 4.83% to 6.04% and 4.89% to 6.23% respectively. After three different pretreatments at 140 °C, the total phytosterol content in RF, MV and RT oil increased by 13.48%, 13.90%, 11.09% (compared to untreated samples), respectively, which proved that the three pretreatment methods can increase the extraction content. For individual phytosterols, the level of β-sitosterol, when treated by RF, MW and RT, increased significantly (compared to untreated samples) by 13.12%, 11.97% and 9.32%, respectively. The β-sitosterol and fucosterol in RF oil (1645.67 mg/kg and 705.75 mg/kg, respectively) were significantly higher than in RT oil (1590.28 mg/kg and 694.68 mg/kg, respectively). However, the increase in the total content of phytosterols for the three pretreatments of TPSO were not significant. In conclusion, a longer nutrient retention period was produced by the rapid conversion of electromagnetic energy to thermal energy in MW and RF heating than for the conventional RT, which was consistent with the data reported by Al Juhaimi et al. [18].

### 3.4. Sensory Characteristics and Volatile Compounds of TPSO

**Figure 3:**
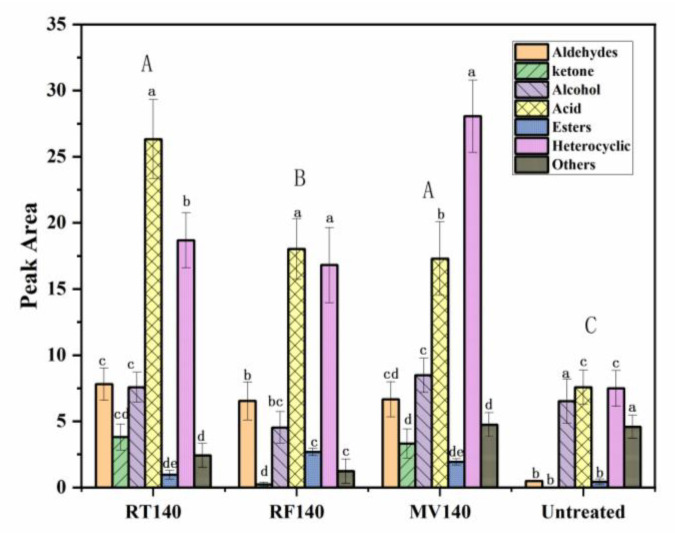
The content of volatile compounds treated by RT, RF, MV pretreatments at 140 °C. Different superscript letters indicate significant differences within the same pretreatment (lowercase, a) and different pretreatments (uppercase, A), respectively (one-way ANOVA and Duncan’s test, *p* ≤ 0.05). RF, radio frequency; MW, microwave; RT, roasting.

Volatile components are also important indicators for evaluating vegetable oils [29,43]. The volatile components of TPSO were significantly enhanced through RF, MW and RT treatment at 140 °C (Supplementary Table S2), increases were determined for mainly aldehydes, ketones, esters, and heterocyclic compounds. In the untreated TPSO, 3-octanol and 1-octen-3-ol, (*Z*)-2-penten-1-ol were detected. According to previous studies, these compounds only appear in untreated oil and are easily destroyed at a high temperature, mainly due to the thermal decomposition of linoleic acid methyl hydrogen peroxide [44]. Figure 3 shows that the total amount of volatile components was lowest with RF treatment; more heterocyclic substances were produced by MW and RT produced more ketone substances. The most dominant heterocyclic substances were 4,7-dimethylbenzofuran and 2,6-dimethylpyrazine, which were significantly increased under RF, MW and RT treatment at 140 °C (*p* < 0.05). Previous research indicated that pyrazines contribute to the roasted aroma [15]. Some alcohols, phenol, and acids were also detected in the TPSO sample of different treatments. The presence of benzyl alcohol and maltol increased with RT and RF treatment. RF and MW were conducive to an increase in lipids, especially γ-butyrolactone (caramel, sweet). Xiao et al. [29] revealed that volatile components in camellia seed oils mainly occurred due to the oxidation of lipase. Furthermore, 2-Furanmethanol increased significantly after pretreatment and was the main alcohol after processing. Benzoic acid increased significantly with RT, providing an unfavorable contribution to the flavor, which was described as pungent (Table S2). The level of benzoic acid in TPSO was very high, and found to be the active constituent, providing the acaricidal activity of the peony root bark [45]. Acetic acid also increased significantly for RF, MV, RT at 140 °C, this substance was also detected in palm kernel oil and Camellia seed oil. It is generated at an early stage in the Maillard reactions [46], and in this study we observed that the content increased most dramatically after RT treatment at 140 °C.

**Figure 4:**
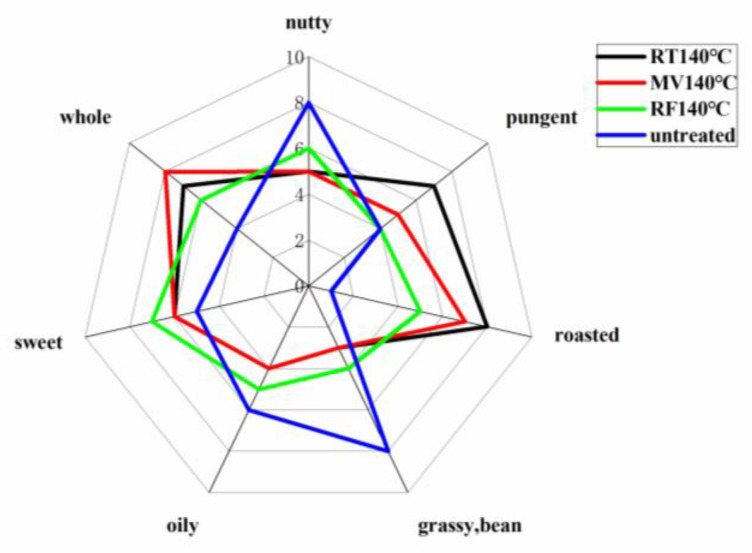
Sensory evaluation treated by RT, RF, MV pretreatments at 140 °C. RF, radio frequency; MW, microwave; RT, roasting.

According to Figure 4, the TPSO scored higher as being grassy and nutty, which are classified as positive sensory attributes, and pungency was reported as an aftertaste in TPSO. This may be caused by the slight oxidation of TPSO due to a high α-linolenic acid level (≥45.53%), and by benzoic acid and other phenolics in TPSO. After RF, MW, and RT treatments at 140 °C, the roasted aroma was found to be strong, the overall aroma increased, the grassy aroma was masked, and nutty and oily characteristics weakened. This also verifies the results of GC-MS measurements, as the heterocyclic substances considerably increased after three treatments. At the same temperature, the volatile aroma component of the RF sample was weaker than that of MW and RT, whereas the scent of the MW samples was gentler than that of the RT samples. This may be due to the differences in molecular vibration [12,21]. The actual mechanism for these differences is unclear.

### 3.5. Effect of Pretreatment on the Antioxidant Capacities and Oxidative Stabilities of Oils

Firstly, the antioxidant capacities of TPSO were evaluated through the free radicals’ scavenging ability (DPPH and FRAP). Table 3 shows that the in vitro antioxidant properties of TPSO, after being treated with different RF temperatures, significantly increased. The extract of oil produced by the untreated seed was able to remove NO·free radicals and the removal rate was very small (DPPH) compared to other vegetable oils [4,14,18]. The DPPH and FRAP scavenging ability of the untreated sample were 2.65 and 27.69 μmol TE/100 g, respectively, which significantly increased (33.26–26.40 μmol TE/100 g DPPH and 65.84–82.54 μmol TE/100 g FRAP) with different RF temperature treatments. Radical scavenging increased significantly after different RF temperatures. The antioxidant effect was significantly increased with RF120 °C compared to the untreated sample (from 2.65 to 23.82 μmol TE/100 g). To better compare the different thermal based pressing methods under same operating conditions, antioxidation was tested during the pressing process, the results for which are shown in Table 3. The DPPH and FRAP values of TPSO, regardless of the use of RF, MW or RT heating pretreatments at 140 °C, increased compared to the values for the untreated groups. FRAP and DPPH scavenging abilities increased by 2.37, 2.98 and 2.42 times/12.55, 12.22 and 9.96 times, respectively. RF, MW and RT treatments produced excellent antioxidant properties in the resulting oi. These results may be caused by the substantial increase in the content of TPC [47]. FRAP and DPPH were associated with reducing the power of an antioxidant, and their increase in the TPSO implies an increased ability to quench free radicals in a system [48]. Therefore, increased radical scavenging could be explained by the possible formation of reducing compounds.

The correlation analysis results of its main bioactive compound (Table 4) show that DPPH, FRAP and the main bioactive compounds in TPSO at RT, MW, RF at 140 °C were significantly correlated at a 0.01 level or 0.05 level. Different heating treatments increased the bioactive compounds and enhanced the oxidation stability of TPSO. Regardless of the pretreatment, positive correlations were observed between the antioxidant activity, and the levels of γ-tocopherol, β-sitosterol, fucosterol, squalene and TPC. More specifically, DPPH was found to be highly correlated with TPC (r = 0.955, *p* < 0.05), γ-tocopherol content, (r = 0.965, *p* < 0.05), β-sitosterol levels (r = 0.996, *p* < 0.05), fucosterol content (r = 0.925, *p* < 0.01), and squalene content (r = 0.902, *p* < 0.01) suggesting that most of the antioxidant activity could be attributed to these components. In addition, FRAP was highly correlated (*p* < 0.05) with TPC (r = 0.986) and β-sitosterol (r = 0.960), followed by γ-tocopherol, fucosterol and squalene (r = 0.932–0.895; *p* < 0.01). In general, highly positive correlations between bioactive properties and the antioxidant capacities of raw samples and those treated using an RF, MV or RT pretreatment at 140 °C demonstrated the excellent contribution of these compounds to the bioactivity and functional potentials of TPSO.

POV, CD, CT, and P-AV were determined as indicators of the oxidation of lipids during the treatment process. The primary oxidation products, POV and CD, increased with an increasing RF heating temperature, from 0.29 to 0.42 mmol/kg and from 1.24 to 1.65, respectively. An increase in temperature caused the production of hydrogen peroxide, which increases the hydrolytic rancidity [10]. The POV of TPSO of untreated and heat-treated seeds ranged between 0.29 and 1.54 mmolO_2_/kg, and the values significantly differ from each other. In contrast, oxidation stabilization effect of MW pretreatment was more pronounced. The increase in POV may be due to the high-temperature oxidation used in the heating treatment. However, Ghafoor et al. [49] studied the POV of poppy seeds, and the oil had similar values to those obtained in this study, but its antioxidant capacity (DPPH) and TPC content were reduced after RT and MV pretreatment. Their results contradict those of this study, which may be a result of differing materials and technology conditions. Unexpectedly, there were insignificant changes in the CT values of the analyzed oil samples independent of the RF heating temperature of tree peony; the value ranged from 0.16–0.24. Conversely, the CT amounts of TPSO were found to be insignificantly different between MW (lowest 0.16) and RT (highest 0.25). P-AV was the measurement of the secondary oxidation product of aldehydes and ketones. The P-AV ranged from 1.19 to 1.49, which showed significant differences (*p* < 0.05) between the three different pretreatments. P-AV values were 1.26, 1.49, and 1.43 after different pretreatment with RF, MW, and RT respectively. MW and RT had the highest values, caused by the oxidative degradation of linoleic acid (abstraction C11 and C9), the Maillard reaction, Strecker degradation, and oxidation processes, which result in the formation of heterocycles, ketones, and aldehydes, and decompose the hydroperoxides, resulting in the formation of aldehydes and ketones [50]. The results are consistent with the previously determined volatile compounds. In addition, the IP value of untreated TPSO was found to be 1.07 h, mainly caused by the high content of unsaturated fatty acids. the value significantly increased to 4.33 h, 4.53 h and 3.11 h after RF, MW and RT treatments at 140 °C, respectively.

## 4. Conclusions

In this study, the effects of RF pretreatment at three different temperatures on the quality in TPSO and its comparison with MW and RT pretreatments at 140 °C were investigated. The results demonstrate that the RF pretreatment could significantly improve TPSO qualities, since it enhances oil yield, increases bioactive compounds and it improves flavor and the oxidation resistance. TPSO was found to have improved qualities when the seed was treated by RF at 140 °C. RF can be applied to the processing of TPS, effectively destroying its internal structure and increasing the oil yield (15.23%). It significantly affected the TPC and antioxidant (DPPH and FRAP) capacities of TPSO (*p* < 0.05), but the flavor was not as strong as the oil produced by MW and RT pretreatments. These findings may serve as a reference for improving the application of RF technology to oilseeds. In addition, RF, MW, and RT treatments at 140 °C significantly affected the characteristic compositions compared to untreated TPSO (the phytosterol increased by 331.06, 341.35 and 272.37 mg/kg and tocopherol increased by 51.63, 35.40, and 18.43 mg/kg, respectively). RF pretreatment could more effectively enhance the bioactive compounds of the oil compared with RT, thereby increasing their nutritional value. By comparing all three pretreatments, we found that the strongest antioxidant activity was produced by MW. The primary oxidation POV and CD were enhanced further by RF; the secondary oxidation products of the P-AV and CT showed no significant changes (*p* < 0.05). In conclusion, the three preheating treatments not only induce the rapid extraction of edible oil but also improve the quality of TPSO. Deacidification and the mechanism of producing volatile compounds after different pretreatments should be further considered for TPSO production. These results can provide guidance for the industrial applications of TPSO. 

## Figures and Tables

**Table 1:** Acid values, colour, oil yield, total phenolic content and total carotenoids of TPSO.

Treatment	Acid Value (mg/g)	L	a*	b*	Oil Yield (%)	TPC (mgGAE/100 g)	Total Carotenoids (mg/kg)
Untreated	4.07 ± 0.04 ^a^	64.81 ± 0.23 ^a^	−4.22 ± 0.13 ^a^	91.52 ± 0.40 ^e^	15.57 ± 013 ^a^	4.78 ± 0.01 ^a^	0.41 ± 0.02 ^a^
RF80 °C	5.14 ± 0.01 ^b^	65.20 ± 0.11 ^a^	−1.24 ± 0.13 ^b^	91.42 ± 0.62 ^e^	25.59 ± 0.11 ^b^	28.78 ± 0.21 ^b^	0.47 ± 0.02 ^a^
RF100 °C	5.21 ± 0.00 ^b^	65.82 ± 0.44 ^a^	2.73 ± 0.52 ^c^	91.19 ± 0.28 ^e^	25.58 ± 0.07 ^b^	33.86 ± 1.57 ^c^	0.47 ± 0.02 ^a^
RF120 °C	6.31 ± 0.03 ^c^	65.03 ± 0.29 a^b^	6.53 ± 0.08 ^d^	95.48 ± 0.35 ^d^	27.61 ± 0.05 ^c^	50.62 ± 2.85 ^d^	0.47 ± 0.02 ^a^
RF140 °C	6.87 ± 0.01 ^d^	64.32 ± 0.29 ^b^	7.67 ± 0.33 ^e^	104.34 ± 0.33 ^b^	30.79 ± 0.11 ^d^	54.12 ± 0.06 ^e^	0.62 ± 0.15 ^a^
MW140 °C	6.65 ± 0.03 ^d^	63.48 ± 0.50 ^c^	11.79 ± 0.33 ^f^	101.26 ± 0.24 ^c^	31.88 ± 0.13 ^e^	64.31 ± 0.01 ^f^	1.35 ± 0.18 ^b^
RT140 °C	6.91 ± 0.02 ^d^	62.32 ± 0.27 ^d^	12.10 ± 0.01 ^f^	105.69 ± 0.07 ^a^	30.27 ± 0.21 ^d^	61.88 ± 0.93 ^f^	0.71 ± 0.02 ^a^

Values represent the mean ± SD of triplicates (*p* < 0.05). Different small letters indicate significant differences (*p* < 0.05) within a row. CIE L*a*b* color system (L* luminosity; a* red/green coordinate; b* yellow/blue coordinate); TPC, total phenolic content.

**Table 2:** Fatty acid composition, and tocopherols, and phytosterols contents in TPSOs obtained after different pretreatments (RF, RT, MW).

Fatty Acids (%)							
	Untreated	RF80 °C	RF100 °C	RF120 °C	RF140 °C	MW140 °C	RT 140 °C
C16:0	5.640 ± 0.003 ^a^	5.640 ± 0.091 ^a^	5.635 ± 0.008 ^a^	5.648 ± 0.009 ^ab^	5.695 ± 0.003 ^ab^	5.646 ± 0.014 ^ab^	5.731 ± 0.006 ^b^
C16:1	0.334 ± 0.020 ^a^	0.385 ± 0.011 ^ab^	0.365 ± 0.098 ^ab^	0.424 ± 0.030 ^ab^	0.381 ± 0.018 ^ab^	0.463 ± 0.062 ^b^	0.372 ± 0.034 ^ab^
C18:0	1.316 ± 0.045 ^a^	1.275 ± 0.065 ^a^	1.242 ± 0.008 ^a^	1.297 ± 0.004 ^a^	1.282 ± 0.018 ^a^	1.256 ± 0.000 ^a^	1.300 ± 0.008 ^a^
C18:1	22.687 ± 0.078 ^bc^	22.580 ± 0.296 ^abc^	22.608 ± 0.004 ^abc^	22.824 ± 0.093 ^bc^	22.911 ± 0.018 ^c^	22.327 ± 0.105 ^a^	22.590 ± 0.042 ^abc^
C18:2	24.011 ± 0.380 ^a^	24.104 ± 0.062 ^ab^	24.655 ± 0.064 ^c^	24.291 ± 0.025 ^abc^	24.414 ± 0.041 ^bc^	24.426 ± 0.078 ^bc^	24.258 ± 0.002 ^ab^
C18:3	46.066 ± 0.501 ^c^	45.919 ± 0.333 ^bc^	45.445 ± 0.012 ^ab^	45.467 ± 0.081 ^abc^	45.270 ± 0.027 ^a^	45.883 ± 0.107 ^bc^	45.750 ± 0.079 ^abc^
SFA	7.288 ± 0.028 ^a^	7.298 ± 0.166 ^a^	7.246 ± 0.081 ^a^	7.368 ± 0.035 ^a^	7.357 ± 0.033 ^a^	7.365 ± 0.076 ^a^	7.403 ± 0.036 ^a^
UFA	92.763 ± 0.042 ^b^	92.602 ± 0.025 ^a^	92.707 ± 0.080 ^ab^	92.585 ± 0.037 ^a^	92.595 ± 0.033 ^a^	92.635 ± 0.076 ^a^	92.598 ± 0.035 ^a^
Tocopherols(mg/kg)							
α-Tocopherols	51.76 ± 0.78 ^d^	49.21 ± 0.79 ^bcd^	44.92 ± 4.39 ^ab^	43.64 ± 0.48 ^a^	45.96 ± 1.44 ^abc^	49.99 ± 0.20 ^cd^	44.57 ± 0.97 ^a^
γ-Tocopherols	714.61 ± 4.66 ^a^	717.64 ± 2.85 ^ab^	733.58 ± 12.50 ^bc^	748.56 ± 0.39 ^cd^	778.76 ± 11.45 ^e^	761.75 ± 2.96 ^d^	750.44 ± 1.13 ^d^
σ-Tocopherols	6.40 ± 0.05 ^b^	6.26 ± 0.03 ^ab^	6.37 ± 0.05 ^b^	6.60 ± 0.03 ^b^	9.51 ± 0.28 ^c^	5.63 ± 0.49 ^a^	6.32 ± 0.42 ^b^
Total	785.26 ± 10.28 ^a^	795.43 ± 24.55 ^a^	820.24 ± 16.29 ^a^	781.70 ± 27.64 ^a^	836.71 ± 13.19 ^a^	820.02 ± 3.50 ^a^	803.61 ± 1.68 ^a^
Phytosterols(mg/kg)							
Squalene	13.82 ± 0.30 ^a^	14.45 ± 0.47 ^ab^	14.36 ± 0.59 ^ab^	15.22 ± 0.17 ^bc^	15.62 ± 0.58 ^cd^	16.58 ± 0.57 ^d^	15.16 ± 0.25 ^bc^
Campesterol	30.30 ± 0.48 ^a^	30.63 ± 0.54 ^ab^	30.98 ± 0.36 ^ab^	30.78 ± 0.76 ^ab^	32.02 ± 0.21 ^bc^	32.87 ± 0.39 ^c^	33.035 ± 1.23 ^c^
γ-Stigmasterol	8.61 ± 0.85 ^a^	9.90 ± 0.47 ^b^	9.73 ± 0.57 ^b^	10.43 ± 0.43 ^b^	10.30 ± 0.02 ^b^	10.62 ± 0.11 ^b^	10.04 ± 0.24 ^b^
β-Sitosterol	1454.75 ± 4.16 ^a^	1558.20 ± 4.92 ^bc^	1532.77 ± 4.74 ^ab^	1575.57 ± 2.76 ^bcd^	1645.67 ± 3.03 ^d^	1628.88 ± 0.43 ^cd^	1590.28 ± 3.72 ^bcd^
Fucosterol	677.24 ± 0.97 ^a^	686.41 ± 3.37 ^abc^	680.31 ± 2.48 ^ab^	701.03 ± 2.13 ^cd^	705.75 ± 1.31 ^d^	707.64 ± 2.22 ^d^	694.68 ± 1.21 ^bcd^
Δ-5-Avenasterol	117.98 ± 2.29 ^a^	126.84 ± 2.93 ^a^	159.79 ± 2.17 ^b^	164.56 ± 1.90 ^bc^	167.36 ± 1.51 ^bc^	171.29 ± 0.98 ^c^	164.67 ± 1.89 ^bc^
Δ-7-Avenasterol	33.73 ± 1.32 ^a^	34.69 ± 0.14 ^ab^	38.04 ± 0.74 ^c^	36.64 ± 0.48 ^bc^	37.40 ± 1.15 ^c^	38.14 ± 0.71 ^c^	38.18 ± 0.86 ^c^
24-Methylenecycloartan-3β-ol	119.37 ± 2.26 ^a^	129.19 ± 0.37 ^b^	158.25 ± 2.18 ^c^	178.93 ± 0.98 ^de^	172.74 ± 1.38 ^d^	191.16 ± 2.01 ^f^	182.13 ± 1.85 ^e^
Total	2455.79 ± 44.04 ^a^	2590.29 ± 32.59 ^b^	2624.21 ± 32.86 ^bc^	2713.14 ± 14.16 ^cd^	2786.85 ± 77.75 ^d^	2797.14 ± 7.99 ^d^	2728.16 ± 24.78 ^cd^

Values are means ± standard deviations, *n* = 3 replicates per treatment. Different superscript letters within the same row indicate significant differences (one-way ANOVA and Duncan’s test, *p* ≤ 0.05). RF, radio frequency; MW, microwave; RT, roasting; SFAs, saturated fatty acids; UFAs, unsaturated fatty acids.

**Table 3:** The content of antioxidant capacities and oxidation stability index of TPSO.

	FRAP (umol TE/100 g)	DPPH (umol TE/100 g)	CD	CT	POV (mmol O_2_/kg)	P-AV	IP(h)
Untreated	27.69 ± 1.05 ^a^	2.65 ± 0.13 ^a^	1.24 ± 0.005 ^a^	0.16 ± 0.003 ^a^	0.29 ± 0.01 ^a^	1.20 ± 0.11 ^a^	1.07 ± 0.12 ^a^
RF80 °C	42.56 ± 0.88 ^b^	3.81 ± 0.31 ^a^	1.31 ± 0.013 ^a^	0.16 ± 0.005 ^a^	0.35 ± 0.01 ^a^	1.23 ± 0.08 ^a^	1.21 ± 0.08 ^a^
RF100 °C	79.41 ± 1.86 ^c^	5.51 ± 0.45 ^b^	1.49 ± 0.021 ^a^	0.19 ± 0.009 ^a^	0.71 ± 0.01 ^b^	1.20 ± 0.12 ^a^	2.14 ± 0.08 ^b^
RF120 °C	89.61 ± 0.69 ^d^	23.82 ± 0.37 ^c^	1.65 ± 0.025 ^a^	0.24 ± 0.003 ^a^	1.07 ± 0.01 ^c^	1.18 ± 0.05 ^a^	3.07 ± 0.11 ^c^
RF140 °C	93.53 ± 2.36 ^e^	35.91 ± 0.15 ^e^	2.21 ± 0.048 ^b^	0.23 ± 0.007 ^a^	1.42 ± 0.01 ^d^	1.26 ± 0.11 ^a^	4.33 ± 0.13 ^d^
MW140 °C	110.23 ± 2.04 ^f^	35.04 ± 0.05 ^e^	1.35 ± 0.027 ^a^	0.16 ± 0.002 ^a^	1.13 ± 0.01 ^c^	1.49 ± 0.05 ^a^	4.53 ± 0.15 ^d^
RT140 °C	94.59 ± 1.46 ^e^	29.05 ± 0.46 ^d^	1.86 ± 0.008 ^b^	0.25 ± 0.007 ^a^	1.54 ± 0.01 ^d^	1.43 ± 0.03 ^a^	3.11 ± 0.10 ^c^

Values are means ± standard deviations, *n* = 3. Different superscript letters within the same row indicate significant differences (one-way ANOVA and Duncan test, *p* ≤ 0.05). DPPH, 2,2-diphenyl-1-picrylhydrazyl; FRAP, ferric-reducing antioxidant power; CD, conjugated diene; CT, conjugated trienes; POV, peroxide value; P-AV, p-anisidine value; IP, induction period.

**Table 4:** Correlation between main bioactive components and antioxidant capacities of TPSO.

	β-Sitosterol	Fucosterol	Squalene	FRAP	DPPH	TPC
γ-tocopherol	0.982 **	0.967 **	0.895 *	0.900 *	0.965 **	0.846
β-sitosterol		0.944 *	0.915 *	0.960 **	0.996 **	0.929 *
Fucosterol			0.939 *	0.895 *	0.925 *	0.819
Squalene				0.932 *	0.902 *	0.864
FRAP					0.974 **	0.986 **
DPPH						0.955 *

** Significantly correlated at the 0.01 level (two-sided). * Significantly correlated at the 0.05 level (two-sided).

## Supplementary Materials

The following are available online at https://www.mdpi.com/article/10.3390/foods10123062/s1, Table S1: Oil yields and moisture contents of radio frequency (RF) pretreated tree peony seeds samples. Table S2: Odorants and odor descriptors determined by GC−MS for different pretreatments.
