# Supplementary material for: Effects of Radio Frequency Pretreatment on Quality of Tree Peony Seed Oils: Process Optimization and Comparison with Microwave and Roasting
Source: Foods. 2021 Dec 9;10(12):3062. doi: 10.3390/foods10123062 (PMC8700783; doi:10.3390/foods10123062)
Supplement: Supplementary file 1 [file foods-10-03062-s001.zip › foods-1399478-supplementary.pdf]

Supplementary Materials

Moreover, proper moisture is beneficial to promote oil extracted from the seeds, prolonged exposure to heating irradiation reduced the seed moisture content and directly influenced the amount of oil extracted from the seeds . The oil yield reached 30.79 % when the moisture content was 8 % treated with RF 140 ℃. It was higher than the moisture content of 6 % and 7 % (exceeded by 4.13 %, 3.54 %, respectively). Compared to the moisture content of 8%, the oil yield of the samples (the moisture content of 9 %,10 %) treated with RF 140℃ slightly reduced by 1.03 % and 0.54 %, respectively. However, the oil yield dropped significantly at 160 ℃, the oil was clearly observed darker and smelled burnt, so those conditions were not used for further experiment.

Table S1

Oil yields and moisture contents of radio frequency (RF) pretreated tree peony seeds samples.

Moisture	Oil yield (%)
6%	29.52%
7%	29.70%
8%	30.79 %
9%	30.47%
10%	30.62%

Table S2

Odorants and Odor Descriptors Determined by GC−MS for different pretreatment

Compounds	Ordor descripetion	CAS	Peak area			
			untreated	RF140	MW140	RT140
**Alcohol**						
Benzyl alcohol	sweet, flower	000100-51-6	0.4180±0.094	0.7702±0.3214	-	0.3942±0.073
Maltol	caramel	000118-71-8	-	0.4185±0.0972	0.3241±0.1538	0.5432±0.1045
2-Furanmethanol	burnt	000098-00-0	-	2.8589±1.5102	3.7514±1.0142	3.5206±1.001
3-Furanmethanol	-	004412-91-3	1.0191±0.1420	-	-	-
3-methyl-3-Buten-1-ol	whiskey, malt, burnt	003658-77-3	-	-	-	0.1725±0.0198
6-methyl-5-Hepten-2-ol	sweet, oily	001569-60-4	-	-	-	0.2407±0.0193
dimethyl-Silanediol	-	001066-42-8	-	-	-	1.2479±0.6310
5-methyl-2-Furanmethanol	-	003857-25-8	-	0.4896±0.1112	0.5149±0.0921	0.4022±0.0264
Furaneol	caramel	003658-77-3	-	-	0.1174±0.0074	0.5797±0.0098
(Z)-2-Penten-1-ol	green, plastic, rubber	001576-95-0	0.7493±0.1020	-	-	-
3-Octanol	moss, nut, mushroom	000589-98-0	0.2462±0.1921	-	-	-
1-Octen-3-ol	mushroom	003391-86-4	2.9641±0.2397	-	-	-
**Aldehydes**						
Methional	cooked potato	003268-49-3	-	0.3211±0.029		
Benzeneacetaldehyde	fresh flora	000122-78-1	-	1.181±0.0971	0.6945±0.1432	1.1854±0.4520
Furfural	bread, almond, sweet	000098-01-1	-	3.1111±1.4320	3.7782±1.0192	3.3223±0.5729
2,4-Heptadienal（E,E）	nut, fat	004313-03-5	-	0.3284±0.0378	0.2756±0.0111	0.2930±0.0101
methylbutanal	malt	000590-86-3	-	1.1278±0.1429	-	-

Benzaldehyde	almond, burnt sugar	000100-52-7	0.4973±0.0981	0.4724±0.097	-	0.7499±0.1331
Acetoin	butter, cream	000513-86-0	-	-	0.6174±0.1429	1.0586±0.4193
Octanal	green, citrus-like		-		0.0881±0.003	-
Nonanal	smoky	000124-19-6	-	-	-	0.4660±0.067
5-Methyl-2-furaldehyde	almond, caramel, burnt	000620-02-0	-	-	0.7911±0.1439	0.6128±0.1727
α-ethylidene-Benzeneacetaldehyde	-	004411-89-6	-	-	0.2224±0.0193	0.1237±0.0059
2-acetylfuran	balsamic	001192-62-7	-	-	0.5187±0.1970	0.4352±0.1204
**ketone**						
3-methyl-1,2-Cyclopentanedione	-	000765-70-8	-	-	0.3381±0.2017	0.3013±0.1021
2-acetylpyrrole	nut, walnut, bread	001072-83-9	-	-	0.5156±0.0701	0.4973±0.1207
2(3H)-Furanone, dihydro-3-hydroxy-4,4-dimethyl-	-	000079-50-5	-	-	0.5275±0.1240	0.4067±0.2016
2(5H)-Furanone	-	000497-23-4	-	-	0.2219±0.0610	
4-Cyclopentene-1,3-dione	-	000930-60-9	-	-	0.4205±0.1928	
4H-Pyran-4-one, 2,3-dihydro-3,5-dihydroxy-6-methyl-	-	028564-83-2	-	-		2.1699±0.7724
**Acid**						
9-Octadecenoic acid	sweat, cheese	002027-47-6	-	-	0.4889±0.2201	
(E)-9-Octadecenoic acid	-	000112-79-8	-	0.5410±0.1012	0.541±0.192	
Acetic acid	sour	000064-19-7	1.1927±0.1727	3.9714±0.7824	1.7894±0.521	14.3575±3.1954
Benzoic acid	Pungent-like	000065-85-0	6.0983±1.0421	13.5078±2.7254	13.4438±1.9837	11.3745±1.5726
hexyl ester-Formic acid		000629-33-4	-	-	-	0.2436±0.0267
Propanoic acid	pungent, rancid, soy	000079-09-4	-	-	-	0.2410±0.1211
n-Hexadecanoic acid	sweat	000057-10-3	-	-	1.0394±0.9114	0.1109±0.0018
**Esters**						
γ-butyrolactone	caramel, sweet	000109-08-0	0.4361±0.2134	0.7320±0.2143	1.7315±0.1821	1.254±0.0152
methyl benzoate	lettuce, herb, sweet	000093-58-3	-	0.2114±0.0194	0.5321±0.0024	0.4408±0.1003
ethyl benzoate	flower, celery, fruit	000093-89-0	-	0.0214±0.1037	0.3121±0.0826	0.2378±0.1201
Formic acid, 1-methylethyl ester	-	000629-33-4	-	-	0.1063±0.0116	-
**Heterocyclic**			-	-	-	-
2,3-Dimethylpyrazine	roasted, nutty	005910-89-4	-	0.3477±0.052	0.8203±0.2233	0.5378±0.1491
2,6-Dimethylpyrazine	nutty, hazelnut	000108-50-9	-	1.9461±0.6984	3.8369±1.8143	2.8835±1.1729
2,5-Dimethylpyrazine	peanut	000123-32-0	0.1962±0.0132	-	4.832±1.441	-
Ethylpyrazine	nutty, green	013925-00-3	-	0.5243±0.1032	1.2561±0.7624	0.9040±0.0691
2-Ethyl-6-methylpyrazine	potato, roasted	013925-03-6	-	0.574±0.0182	-	0.7270±0.2361
2,3,5-Trimethylpyrazine	potato,roasted	014667-55-1	-	1.0142±0.2103	-	-
Methylpyrazine	roasted	000109-08-0	0.2599±0.1142	3.2124±1.4201	6.863±0.3451	5.7342±1.2373
2-pentylFuran	caramel-like, roasted	003777-69-3	-	-	0.0827±0.0023	0.1393±0.1101
Trimethylpyrazine	roasted, potato, must	014667-55-1	-	-	2.2487±1.0103	1.6710±0.8921
2-Ethyl-5-methylpyrazine	bean, grassy	013360-64-0	-	0.5721±0.0132	1.1722±0.7936	0.6658±0.1392
3-Ethyl-2,5-dimethylpyrazine	roasted, nut	013360-65-1	-	0.668±0.1969	-	0.3818±0.1271
4,7-Dimethylbenzofuran	-	028715-26-6	7.0345±1.2410	4.6791±2.1938	1.7981±1.0289	3.8794±1.5921

5-methylfurfural	almond, caramel	000620-02-0	-	0.8722±0.0129	-	-
2,3-Dihydro-3,5-dihydroxy-6-methyl-4(H)-pyran-4-one	-	028564-83-2	-	2.4053±1.0021	3.5408±1.3207	3.8794±0.1972
2-Acetyl pyrrole	sweet, nutty	001072-83-9	-	0.5215±0.1935	-	-
Pyridine	rancid	000110-86-1	-	-	0.2205±0.9281	-
2-methylpyridine	sweat	000109-06-8	-	-	0.0732±0.0192	-
Pyrazine	-	000290-37-9	-	-	0.4344±0.0419	0.3973±0.0911
Acetylpyrazine	roast	022047-25-2	-	-	0.1095±0.0021	-
diethylmethyl pyrazine	potato, meat, roast	018138-04-0	-	-	0.0126±0.0013	-
perillen	wood	000539-52-6	-	-	0.0827±0.0113	-
2-ethenyl-6-methyl-Pyrazine	earth, coffee	013925-09-2	-	-	0.3329±0.9810	-
3,5-diethyl-2-methylpyrazine	baked	018138-05-1	-	-	0.3176±0.1725	-
**Others**						
4-ethylcumen	-	004218-48-8	-	0.4517±0.2195	-	-
D-Limonene	citrus, mint	005989-27-5	0.1975±0.0120	0.1243±0.1927	0.1105±0.0192	0.2484±0.0124
Styrene	balsamic, gasoline	000100-42-5	0.6344±0.1327	-	-	0.4564±0.2017
1,3-dimethylbenzen	plastic	000108-38-3	0.2949±0.043	-	-	-
(E)-2-hexenal	apple, green	006728-26-3	2.5574±1.197	-	-	-

Values are means ± standard deviations, n = 3.

Odor is obtained according to the website http://www.flavornet.org/flavornet.html. Compounds: MS, identified by the NIST 17 mass spectral database;

'-' means it is not detected or qual< 80 or not detected
